# A Model Chain Application to Estimate Mixing Layer Height Related to PM10 Dispersion Processes

**DOI:** 10.1155/2015/298492

**Published:** 2015-11-05

**Authors:** F. Guarnieri, F. Calastrini, C. Busillo, G. Messeri, B. Gozzini

**Affiliations:** ^1^LAMMA Consortium, Via Madonna del Piano 10, 50019 Sesto Fiorentino, Italy; ^2^IBIMET, National Research Council, Via G. Caproni 8, 50145 Florence, Italy

## Abstract

The mixing layer height (MLH) is a crucial parameter in order to investigate the near surface concentrations of air pollutants. The MLH can be estimated by measurements of some atmospheric variables, by indirect estimates based on trace gases concentration or aerosol, or by numerical models. Here, a modelling approach is proposed. The developed modelling system is based on the models WRF-ARW and CALMET. This system is applied on Firenze-Prato-Pistoia area (Central Italy), during 2010, and it is compared with in situ measurements. The aim of this work is to evaluate the use of MLH model estimates to characterize the critical episodes for PM10 in a limited area. In order to find out the meteorological conditions predisposing accumulation of PM10 in the atmosphere's lower level, some indicators are used: daily mean wind speed, cumulated rainfall, and mean MLH estimates from CALMET model. This indicator is linked to orography, which has important consequences on local weather dynamics. However, during critical events the local emission sources are crucial to the determination of threshold exceeding of PM10. Results show that the modelled MLH, together with cumulative rainfall and wind speed, can identify the meteorological conditions predisposing accumulation of air pollutant at ground level.

## 1. Introduction

Atmospheric pollution in densely inhabited areas is a matter deeply analysed by scientific community. In particular, the study of atmospheric stability is of primary importance in the evaluation of atmospheric pollution dynamics in those sites. The relationship between mixing layer height (MLH) and air pollution accumulation in the near surface has been investigated in numerous works [1–5]; however the MLH is often a rather unspecific parameter whose definition and estimation are maoften difficult [6].

The MLH can be estimated in different ways: by using measurements of temperature profiles, vertical profiles of the potential temperature, wind speed, humidity, or aerosol concentrations, from radiosonde or remote sensing instruments [4, 7–11]. An indirect estimate of the MLH can be done using empirical methods, based on trace gases concentration as radon [12, 13]. Numerous studies indicate that MLH is directly linked to radon concentrations; in fact the emissions of this gas can considerably change in different areas, but in a limited zone the internal variations depend only on meteorological conditions and particularly on MLH [10].

Another way to estimate MLH is using the numerical models [8, 14, 15]. In particular, the diagnostic meteorological model CALMET has been successfully used to obtain MLH estimates [7, 12, 16, 17]. This method takes the advantage of limited costs, even if it needs a validation process using a dataset of measurements. The numerical models have the relevant advantage that they can be used also in an operational configuration, in such a way to forecast the MLH dynamic. This could be a valid instrument to support air quality monitoring and management.

## 2. Materials and Methods

### 2.1. Overview of the Study Area

**Figure 1:**
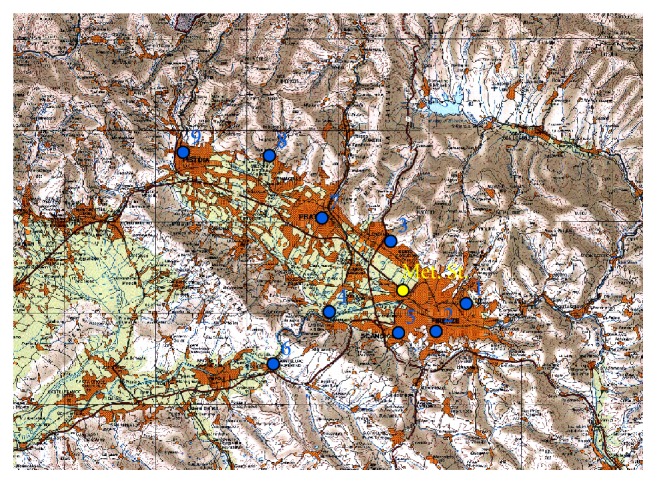
The study area overview with location of ARPAT urban background air quality stations and meteorological station of Firenze-Peretola. See Table 1 for stations metadata.

Figure 1 shows an overview of the study area, located in Tuscany region in the central part of Italian Peninsula. This target zone includes the Florence metropolitan area and the cities of Prato and Pistoia. The study area is the most populated Tuscany area and it contains different emission sources, such as both small and large industries, the Florence international airport, and some major arterial roads. In the Firenze-Prato-Pistoia area are located nine air quality urban background stations of the ARPAT (Environmental Protection Agency of Tuscany Region) monitoring network [18] and one synoptic weather station within the WMO monitoring network, named Firenze-Peretola.

In order to characterize the critical pollution episodes in the target area occurring during 2010, the PM10 data from ARPAT monitoring network are used. The mean daily PM10 concentrations are determined by the gravimetric method for the nine background stations, whose metadata are shown in Table 1. In this table is also shown the information about the WMO synoptic station where wind speed, temperature, and precipitation are collected.

### 2.2. The Modelling System

The developed model system is based on the prognostic model WRF-ARW [19], which provides the meteorological input fields to the diagnostic model CALMET [14]. The WRF-ARW model is initialized and forced at the boundaries every 6 hours by ECMWF analysis dataset [20]. A two-nested grid layout is chosen: the coarsest domain with horizontal resolution of 9 km, covering the whole Italian Peninsula, the finest domain with 3 km grid resolution focussing on the North-Central Italy. This configuration has 35 sigma vertical levels with a stretching factor to obtain a higher resolution near the surface (the first level is approximately 20 meters).

**Figure 2:**
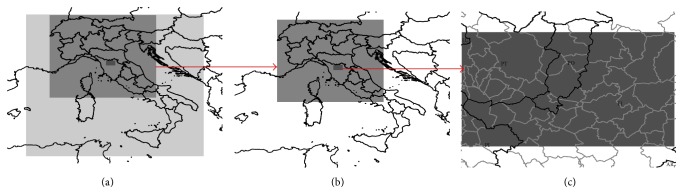
Modelling system configuration: WRF-ARW 9 km domain (a), WRF-ARW 3 km domain (b), and CALMET 500 m domain (c).

The meteorological field data from WRF-ARW simulations provide the input data for the diagnostic CALMET model. CALMET is configured on a smaller domain covering the Firenze-Prato-Pistoia area, with a finer resolution of 0.5 km, and it operates in a terrain-following vertical coordinate system with 14 vertical levels from 10 to 4500 meters. This modelling setting is shown in Figure 2.

**Figure 3:**
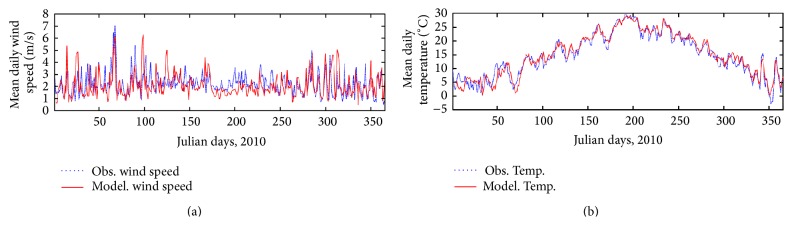
Daily mean wind speed (a) and mean temperature (b) simulated by CALMET model (blue line) and observed at Firenze-Peretola station (red line), for the whole 2010.

The modelling system is applied to simulate a whole meteorological year, in particular the 2010 year. The results are compared to in situ observations. In particular, the daily mean wind speed and temperature, from WMO synoptic weather station Firenze-Peretola, are considered for model validation. This comparison shows a good agreement between simulations and observations, as reported in Table 2, in which some of the most used skill scores are evaluated (correlation, mean bias, index of agreement, mean normalised gross error, and normalised mean bias).  Figure 3 shows the daily mean wind speed and daily mean temperature, considering Firenze-Peretola station and the CALMET grid point closest to this observation location.

The MLH has been simulated by CALMET model using Gryning-Batchvarova algorithm [21], and it is expressed in meters above the ground level. The validation of this modelling method has been carried out in a previous work using hourly radon concentration measurements collected by University of Florence, in the framework of an extensive field campaign from 09/05 to 06/06. The correlation between hourly radon concentration and MLH model estimate showed a good agreement from both hourly and daily point of view, confirming a proper configuration of modelling system on Firenze-Prato-Pistoia area [22, 23].

## 3. Results and Discussion

### 3.1. Meteorological Characterization

This paragraph discusses meteorological characterization of the target area of Firenze-Prato-Pistoia, based on modelled temperature, wind speed, and MLH obtained by the WRF-CALMET system.

**Figure 4:**
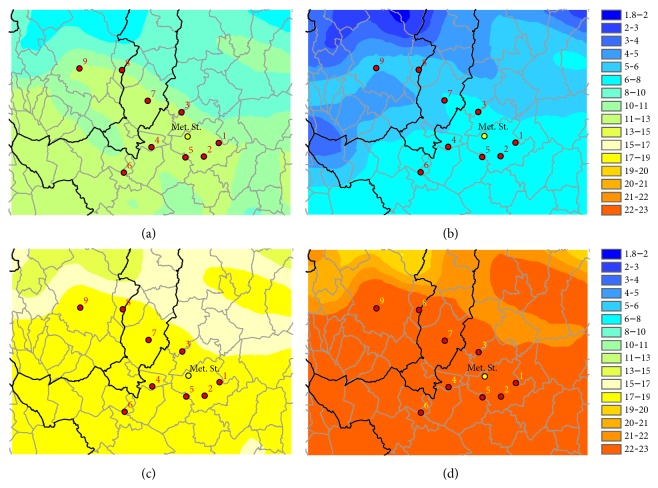
WRF-CALMET mean temperature (°C) for the four seasons on the simulation period, for the inner domain: autumn (a), winter (b), spring (c), and summer (d).

The mean annual temperature in the study area is rather homogeneous, with values between 13 and 15°C, with minimum in mountain area and maximum around 15–17°C in the inner area around Florence. During spring and summer the temperature in the Firenze-Prato-Pistoia target area is homogeneous, while in winter and autumn there is difference of around 2 degrees between Pistoia and Prato area with respect to the warmer Florence area (Figure 4).

**Figure 5:**
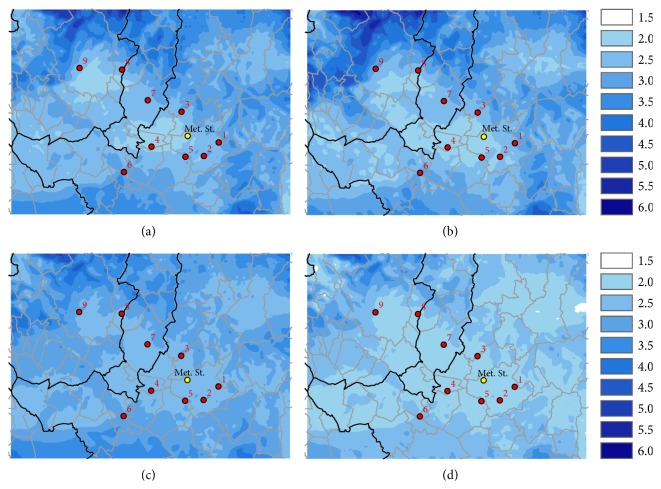
WRF-CALMET mean wind speed (m/s) for the four seasons on the simulation period, for the inner domain: autumn (a), winter (b), spring (c), and summer (d).

The mean annual wind speed records higher values over hills and mountains, while in the area there are two minimums in the southern part of Pistoia and in the area between Prato and Florence, with values around 2 meters per second. The spring is the windiest season, and summer is less windy with values ranging between 1.5 and 2 meters per second. During spring and summer, the values are rather homogeneous in the target area, while during autumn and winter the southern part of Pistoia and the area between Prato and Florence are characterized by lower wind with respect to the global domain (Figure 5).

**Figure 6:**
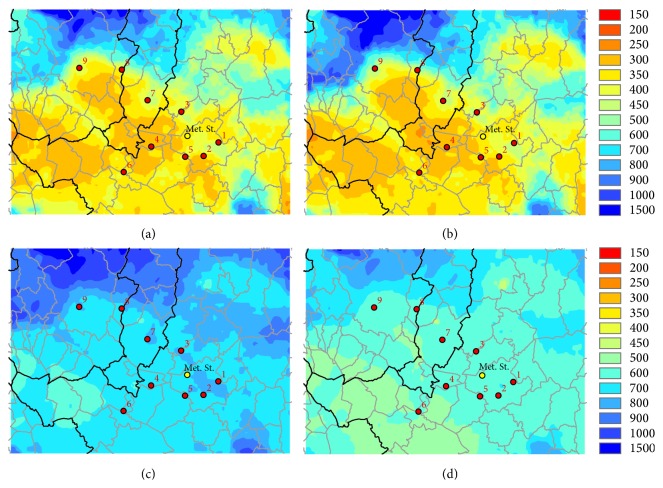
WRF-CALMET mean MLH (m) for the four seasons on the simulation period, for the inner domain.

The annual mean MLH values trace the orography profile, with higher values over mountains, similarly to the wind speed. The spring and summer seasons record higher values in the study area, while during autumn and winter seasons there are very low values on the study area. Furthermore, during autumn and winter, in the inner part of the target area there are scattered values, with a minimum in the central part of the domain, as shown in Figure 6. These MLH's variations in the inner part of the Firenze-Prato-Pistoia area, although relatively modest, are certainly caused by turbulence, as it can be noticed comparing the map of wind speed and MLH.

**Figure 7:**
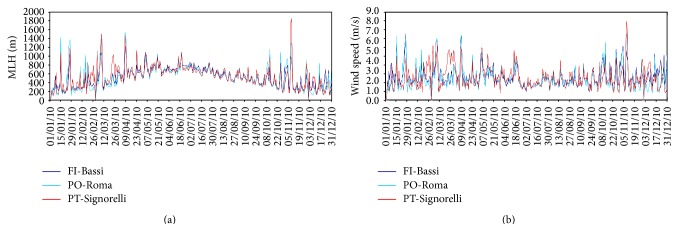
Time series of simulated MLH (a) and wind speed (b) extracted in correspondence of FI-Bassi, PO-Roma, and PT-Signorelli air quality stations.

The time series of modelled MLH evaluated in the grid cells corresponding to the position of the nine urban background stations located in the target area are highly correlated with each other for the whole 2010, with correlation coefficients around 0.95–0.98. This fact shows that in the target area there is a good accordance among the modelled time series of MLH in terms of time trend. Some considerations here reported are conducted based on a subset of only three stations, in particular FI-Bassi, PO-Roma, and PT-Signorelli, which are representative for the three main cities areas. In Figure 7, the mean daily modelled MLH time series corresponding to the chosen air quality stations are shown for the entire simulation period. It can be noticed that, during warm season, when solar radiation reaches its maximum, the mean value of MLH is higher than in the cold season. It is important to underline that during autumn and winter seasons MLH time series shows some frequent high-level peaks: these values occur during meteorological events characterized by strong winds, as shown comparing time series of Figure 7.

**Figure 8:**
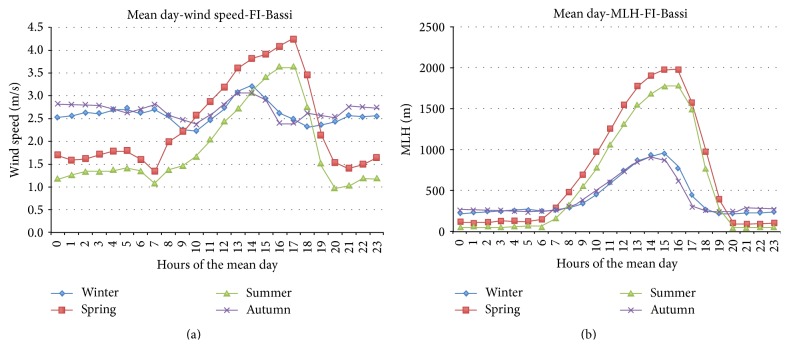
Mean day of modelled wind speed (a) and modelled MLH (b) in correspondence of FI-Bassi station, for all seasons.

The estimates of MLH and the estimates of wind speed are elaborated in order to obtain a “mean day.” The seasonal mean day shows similar behaviour for all the time series, for both MLH and wind speed. As example, in Figure 8, is represented the mean day relative to modelled wind speed and modelled MLH for FI-Bassi location. During autumn-winter season the mean daily wind shows an almost constant trend, while during spring-summer season there are higher values in the central hours of the day, due to the breeze regimes. The mean daily MLH during spring-summer season shows higher values in the daytime with respect to autumn-winter season, related to the radiative forcing.

### 3.2. Analysis of PM10 Concentrations Related to MLH

**Figure 9:**
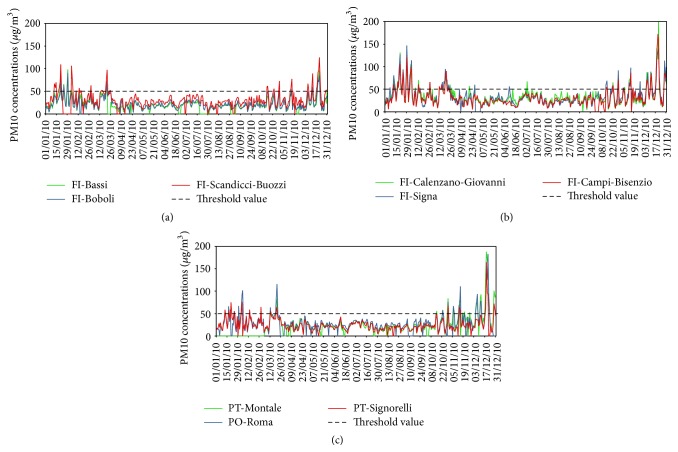
Time series of observed PM10 for the nine air quality stations in the study area: (a) FI-Bassi, FI-Boboli, and FI-Scandicci, (b) FI-Calenzano, FI-Signa, and FI-Campi-Bisenzio, and (c) PT-Montale, PO-Roma, and PT-Signorelli.

In this paragraph the time trends of PM10 concentrations related to the estimate of MLH are analysed. The time series of PM10 relative to the urban background stations in the target area are highly correlated with each other, showing a good accordance among them in terms of time trend, for the whole 2010. The correlation coefficients are around 0.8-0.9. The whole set of monitoring stations registers the maximum concentration values during the cold season, highlighting the same critical episodes, even if some substantial differences occur from quantitative point of view (see Figure 9). This is particularly important when the concentration levels of PM10 exceed the daily and the annual limit values established by European Union (Directive 2008/50/EC), while during the warm season the time series show both time trend and concentration values similar.

**Figure 10:**
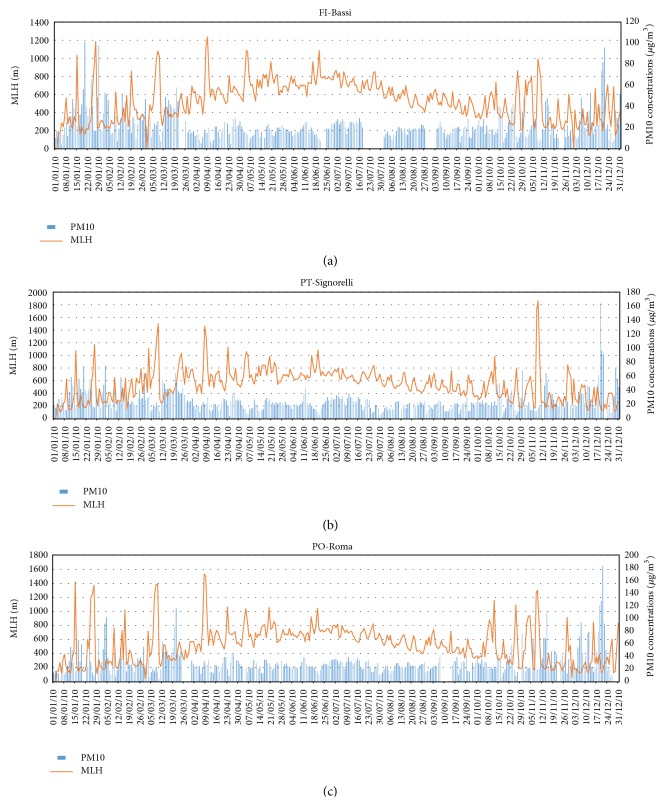
Time series of observed PM10 and MLH estimates for (a) FI-Bassi, (b) PO-Roma, and (c) PT-Signorelli stations.

In Figure 10 are shown the PM10 and the MLH time series in correspondence of FI-Bassi, PO-Roma, and PT-Signorelli stations. It can be noticed that, especially during winter, at high concentrations of PM10 correspond low MLH values, and vice versa. This is confirmed by the correlation's coefficients between MLH and PM10 time series, for the nine ARPAT background stations. In this case an anticorrelation is found between the MLH and PM10 time series, with values ranging from −0.51 to −0.33.

**Figure 11:**
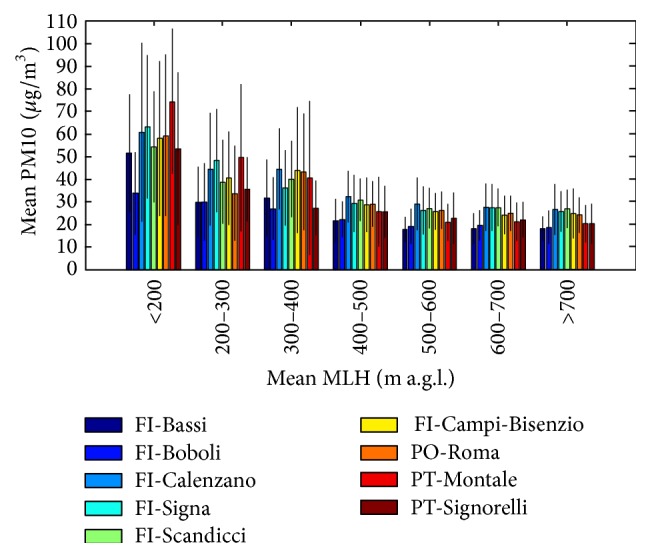
Mean PM10 values and their standard deviations at the nine air quality stations dependent on 7 classes of mean daily MLH for the periods without precipitation or with precipitation lower than 10 mm, during 2010.

Following Holst et al. (2008), a further synthetic description about the influence of MLH on PM10 concentrations is shown in Figure 11. Here, the mean PM10 values at the nine air quality considered stations are represented on dependence on 7 classes of mean daily modelled MLH. The evaluated data are relative to periods with missing precipitation or daily-cumulated precipitation lower than the arbitrary threshold of 10 mm. In fact, as demonstrated in a previous work [23] focussed on the target area, only the precipitation above this threshold has a wash-out effect. The results show a decrease of mean PM10 values in all the considered stations with increasing MLH classes. In particular, the first MLH class (values lower than 200 m) is the most critical, because eight of the nine stations register mean PM10 values above the alarm threshold of 50 *μ*g/m^3^.

In order to find out the meteorological conditions predisposing the accumulation of PM10 in the atmosphere's lower level, the critical episodes occurring in 2010 are identified considering the alarm threshold values of 50 *μ*g/m^3^ for urban background stations in the target area. Based on need to represent a common pollutant condition for the Firenze-Prato-Pistoia area, the episodes that involve the most part of the stations are considered, revealing that the critical days during 2010 are about 60.

The most critical period, as already mentioned, is the cold season, particularly from January to March and from October to December. During January, February, and March, two long-lasting episodes occurred, interrupted only by few short intervals. In December three episodes are registered from 5 to 30. In the second part of February and during October and November the critical periods are more episodic.

In previous works [22, 23] focussed on Tuscany region, some meteorological indicators are identified in order to characterize the meteorological conditions predisposing the accumulation of pollutants in the lower levels of atmosphere: in particular, the daily cumulative rainfall and the daily wind speed, from both simulation and observation data. The critical episodes occurring in 2010 are characterized by a weak wind and very weak or missing precipitation, as already found out in the previous works [23]. The daily mean estimate MLH from high-resolution CALMET model can be used in order to improve the meteorological description on a limited domain, as the Firenze-Prato-Pistoia area. This additional indicator is linked to detailed orography, which has an important consequence on local weather dynamics. During the air pollution events, the estimates of wind speed and MLH are rather low. In Table 3 is reported the selection of air pollution days occurring during 12/10, with PM10 observations at the nine ARPAT air quality stations, the WRF-CALMET wind speed and MLH estimates, extracted in correspondence of the synoptic Firenze-Peretola station.

**Figure 12:**
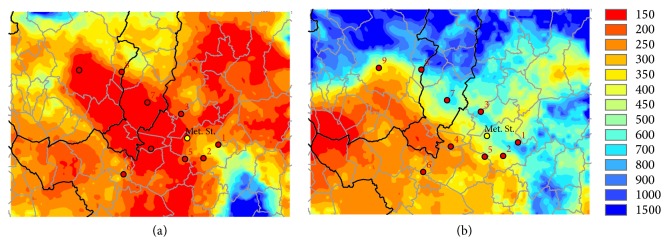
Maps of WRF-CALMET mean daily MLH (m) for 21/12/10 (a) and 27/12/10 (b).

As example, an analysis for two different types of days is here presented: 21/12/10, which is characterized by high atmospheric stability and high PM10 concentrations, with values ranging from 85 to 183 *μ*g/m^3^; 27/12/10, which is characterized by high atmospheric diffusivity due to a persistent northerly wind and very low PM10 concentration values ranging from 8 to 25 *μ*g/m^3^ (see Table 3). In those days the MLH is certainly different, as shown in Figure 12: on 21/12 the values are less than 180 meters and on 27/12 the MLH values are about 600 meters. This confirms the usefulness of MLH model estimates to characterize the critical episodes for PM10 in a limited area.

The analysis of the results shows that the daily maps of MLH obtained through the modelling system simulations, integrated with the information about local meteorological parameters, can identify the meteorological conditions predisposing accumulation of air pollutant at ground level. Finally, the discussed results show that the performed modelling system is robust enough to be implemented in a forecast mode; in fact in next application the high-resolution modelling system will be used in a forecasting configuration, applied to limited areas characterized by complex orography and being densely populated. Tuscany Regional Government will use this instrument to support air quality monitoring and management.

## 4. Conclusions

The aim of the present work is to evaluate the use of MLH model estimates to characterize the critical episodes for PM10 in a limited area. The estimates of MLH, wind speed, and temperature, from WRF-CALMET modelling system in the Firenze-Prato-Pistoia area for the 2010 period, are elaborated in order to obtain mean daily, annual, and seasonal fields. An analysis has been performed in order to identify the critical air pollution events. Then, the anticorrelation between MLH estimates and PM10 data are found out.

The analysis of results shows that the daily maps of MLH obtained through high-resolution simulations, integrated with the information coming from daily cumulative rainfall and daily wind speed, can identify the meteorological conditions predisposing accumulation of air pollutant at ground level. However, during critical events characterized by atmospheric stability and low diffusivity, the local emission sources are crucial to the determination of threshold exceeding of PM10.

The analysis of the results shows that the adopted modelling system is robust enough to be used also in a forecast configuration, providing a valid instrument in supporting the decision makers in the management and planning of local air quality.

## Figures and Tables

**Table 1:** Metadata of ARPAT air quality monitoring stations classified as urban background and metadata of the Firenze-Peretola synoptic weather station.

ID	Name	Location	Address	Lat. (°N)	Lon. (°E)	Data	Network	Valid data (%)
1	FI-Bassi	Firenze	v. Bassi	43.79	11.29	PM10	ARPAT	91
2	FI-Boboli	Firenze	v. Boboli	43.76	11.24	PM10	ARPAT	99
3	FI-Calenzano	Calenzano	v. Giovanni XXIII	43.85	11.19	PM10	ARPAT	100
4	FI-Signa	Signa	v. Roma	43.78	11.10	PM10	ARPAT	98
5	FI-Scandicci	Scandicci	v. Buozzi	43.76	11.19	PM10	ARPAT	93
6	FI-Campi-Bisenzio	Campi-Bisenzio	v. Orly	43.73	11.02	PM10	ARPAT	98
7	PO-Roma	Prato	v. Roma	43.87	11.09	PM10	ARPAT	91
8	PT-Montale	Montale	v. Pacinotti	43.94	11.02	PM10	ARPAT	77
9	PT-Signorelli	Pistoia	v. Signorelli	43.94	10.91	PM10	ARPAT	97
Met. St.	FI-Peretola	Firenze	Peretola Airport	43.81	11.20	Temp., Prec., and wind speed	Synop. WMO	100

**Table 2:** Skill scores (correlation, mean bias, index of agreement, mean normalised gross error, and normalised mean bias) for modelled daily wind speed and modelled daily mean temperature with respect to Firenze-Peretola synoptic weather station data, for the whole 2010.

Skill score	Formula	Wind speed	Mean temperature
CORR	$\frac{\sum_{i=1}^{N}\left(O_{i}-\overset{-}{O}\right)\left(M_{i}-\overset{-}{M}\right)}{\sqrt{\sum_{i=1}^{N}{\left(O_{i}-\overset{-}{O}\right)}^{2}}\sqrt{\sum_{i=1}^{N}{\left(M_{i}-\overset{-}{M}\right)}^{2}}}$	0.46	0.98

MB	$\frac{1}{N}\overset{N}{\underset{i=1}{\sum }}\left(M_{i}-O_{i}\right)$	0.53	0.46

IOA	$1-\frac{\sum_{i=1}^{N}{\left(M_{i}-O_{i}\right)}^{2}}{\sum_{i=1}^{N}{\left(\left|O_{i}-\overset{-}{O}\right|+\left|M_{i}-\overset{-}{O}\right|\right)}^{2}}$	0.68	0.99

MNGE	$\frac{1}{N}\overset{N}{\underset{i=1}{\sum }}\frac{\left|M_{i}-O_{i}\right|}{O_{i}}\times 100\%$	51.96	15.40

NMB	$\frac{\sum_{i=1}^{N}\left(M_{i}-O_{i}\right)}{\sum_{i=1}^{N}O_{i}}$	33.44	3.08

*M*_*i*_ and *O*_*i*_ are values of model and observation at time *i*, respectively, *N* is number of samples, and $\overset{-}{M}$ and $\overset{-}{O}$ are mean values of model and observation time series.

**Table 3:** PM10 concentrations at the nine ARPAT air quality stations, with mean daily modelled wind speed and modelled MLH corresponding to Firenze-Peretola station. Values with bold font are those exceeding the threshold of 50 *μ*g/m^3^.

Date	FI-Bassi	FI-Boboli	FI-Calenzano-Giovanni	FI-Signa	FI-Scandicci-Buozzi	FI-Campi-Bisenzio	PO-Roma	PT-Montale	PT-Signorelli	FI-Peretola Wind speed (m/s)	FI-Peretola MLH (m)
01/12/10	6	6	10	11	12	14	11	18	15	2.2	353
02/12/10	12	11	21	21	18	22	23	21	20	1.5	188
03/12/10	9	7	14	17	14	17	21	20	20	—	—
04/12/10	11	12	17	33	24	19	19	33	18	1.2	179
05/12/10	27	34	**50**	**50**	49	**53**	**54**	**61**	38	1.1	153
06/12/10	48	49	**87**	**63**	**67**	**71**	**71**	**75**	37	1.4	151
07/12/10	36	37	**67**	**87**	**55**	**65**	**94**	**71**	36	2.0	258
08/12/10	20	23	38	45	31	42	46	**50**	36	1.8	215
09/12/10	19	20	28	34	28	27	28	—	22	2.1	230
10/12/10	23	22	48	**52**	35	47	41	49	26	1.9	294
11/12/10	36	35	**72**	**89**	**52**	**84**	**76**	**85**	47	1.2	130
12/12/10	**53**	**60**	**86**	**70**	**89**	**76**	**78**	**93**	45	1.0	174
13/12/10	23	29	36	**53**	38	36	29	36	28	1.7	227
14/12/10	14	16	26	32	29	28	23	28	27	1.7	222
15/12/10	21	18	29	42	28	29	15	40	15	4.2	732
16/12/10	19	21	31	40	33	26	22	32	24	1.9	272
17/12/10	22	33	**54**	**56**	**53**	42	47	**62**	48	2.7	372
18/12/10	41	47	**85**	**69**	**61**	**95**	**79**	**137**	**62**	1.3	232
19/12/10	**74**	**75**	**141**	**124**	**93**	**138**	**121**	**188**	**165**	2.0	263
20/12/10	**82**	**71**	**116**	**132**	**96**	**172**	**127**	**167**	**97**	1.1	130
21/12/10	**96**	**85**	**208**	**139**	**125**	**153**	**183**	**162**	**92**	1.4	172
22/12/10	19	20	**50**	**80**	**62**	**67**	**92**	**80**	—	2.5	364
23/12/10	23	23	38	**56**	38	41	**50**	47	—	3.2	471
24/12/10	8	7	14	16	14	17	17	19	13	2.1	316
25/12/10	15	17	31	33	29	27	23	28	23	1.8	225
26/12/10	6	7	10	14	15	12	8	10	8	2.4	435
27/12/10	8	12	15	25	20	14	11	13	13	3.6	629
28/12/10	36	35	**62**	**55**	42	**55**	—	**54**	32	0.8	120
29/12/10	29	33	**100**	**113**	**52**	**86**	—	**101**	**72**	1.1	138
30/12/10	23	38	**81**	**67**	**53**	**79**	—	**86**	**57**	2.3	328
31/12/10	**52**	39	**62**	**99**	**52**	**77**	—	**92**	45	3.4	544
